# Pneumomediastinum and Mediastinal Hematoma Secondary to Right Brachiocephalic Vein Thrombectomy Mimicking STEMI

**DOI:** 10.1155/2017/2796568

**Published:** 2017-07-18

**Authors:** Prem Shukla, Prudence Dy, Rishi Raj, Sayee Sundar Alagusundaramoorthy, Noel Nivera

**Affiliations:** Department of Medicine, Monmouth Medical Center, Long Branch, NJ 07740, USA

## Abstract

A 50-year-old male with a history of hemodialysis dependent chronic kidney disease presented to our emergency department with acute midsternal crushing chest pain. Patient was diagnosed with acute anterolateral wall Myocardial Infraction due to the presence of corresponding ST segment elevations in EKG and underwent emergent cardiac catheterization which revealed normal patent coronaries without any disease. He continued to have chest pain for which CT of the chest was done which revealed pneumomediastinum with mediastinal hematoma, due to the recent attempted thrombectomy for thrombus in his right brachiocephalic vein.

## 1. Background

It is absolutely imperative to rule out cardiac ischemia in patients who present with chest pain and ST segment elevation on EKG. Emergent cardiac catheterization is the standard of care across all hospitals in the United States. However, once a cardiac etiology is ruled out other diagnosis should be sought. Around 26% of angiography done in acute phase of suspected STEMI has been shown to be normal [1]. The incidence of alternative conditions mimicking STEMI is around 2.3% [2]. Pneumomediastinum is a rare condition that can mimic STEMI. This case report aims to report pneumomediastinum after unsuccessful right brachiocephalic vein thrombectomy mimicking a STEMI.

## 2. Case Presentation

We present the case of a 50-year-old male with a history of hemodialysis dependent end-stage renal disease, HIV on antiretroviral therapy, hypertension, and hyperlipidaemia who presented to our emergency room with stinging nonradiating midsternal chest pain since the afternoon associated with difficulty in breathing. Patient was a former smoker with 29-pack-year smoking history and a former alcoholic. Family history revealed MI in the father at the age of 56. Patient underwent unsuccessful right brachiocephalic vein thrombectomy a day prior to presentation to the emergency room.

At the time of physical examination, blood pressure was 128/97 mmHg, heart rate was 84/minute, respiratory rate was 15/minute, temperature was 99.7 F, and SpO_2_ was 93% in room air. Patient was a moderately built obese male, alert, diaphoretic, and in moderate distress from his chest pain and shortness of breath. Cardiovascular examination revealed normal cardiac sounds, no murmurs/rubs, and no chest wall tenderness. Breath sounds were heard in all lung fields without any added sounds. Peripheral pulses were equal and no delay was noted. His AV Fistula in the right arm had a good palpable thrill. The rest of the exam was unremarkable. There were no signs of brachiocephalic vein thrombosis.

## 3. Investigations

Complete blood count with differential showed normocytic normochromic anemia with haemoglobin of 13.2 mg/dl (normal range 13.5–18 mg/dl), white cell count of 8800/cu mm (normal range 4500–11000/cu mm), and platelets of 253,000/cu mm (normal range 140,000–450,000/cu mm). Coagulation profile done was within normal range with PT of 11.1 seconds (normal range 9.6–12.7), INR of 1.0 (normal range 0.9–1.1), and PTT of 30.0 (normal range 2.5–36.9). Serum electrolytes studies showed elevated serum potassium level 5.7 mEq/L (normal range 3.5–5.5 mEq/L) with normal sodium, chloride, calcium, and bicarbonate levels.

Renal function tests showed elevated BUN 39 mg/dL (normal range 5–21 mg) and serum creatinine of 9.41 mg/dL (normal range 0.60–1.20 mg/dL) with severely decreased estimated GFR 6 mL/min/1.73 m^2^ (normal > 60 mL/min/1.73 m^2^). Liver function tests showed elevated serum alkaline phosphatase 109 U/L (normal range 25–100 U/L) with normal serum ALT and AST.

**Figure 1:**
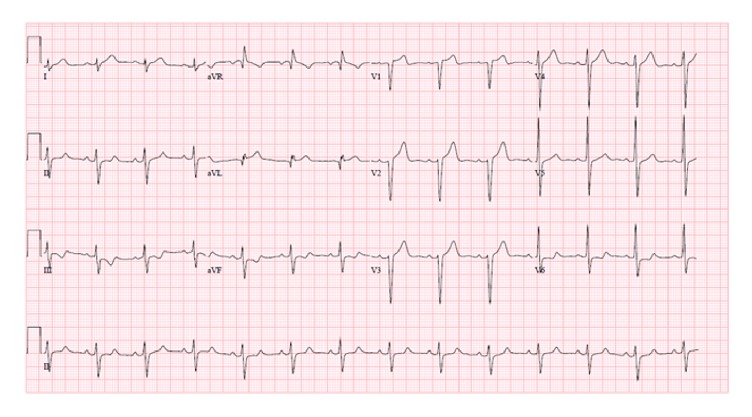
12-lead EKG showing ST elevation changes at V1–V3.

Initial Troponin^−^I done was 0.08 ng/mL (normal: 0.04–0.80 ng/mL). Electrocardiogram (Figure 1) showed normal sinus rhythm, normal axis, and presence of ST elevations in I, aVL, and V1–V4. Also noted were ST depressions in III, aVF, and V6 and incomplete LBBB.

Chest X-ray revealed a small right lower lobe infiltrate with subpulmonic effusion. Echocardiography revealed mild concentric left ventricular hypertrophy, impaired LV relaxation with abnormal (paradoxical) septal motion consistent with LBBB, noncompaction of ventricular apex, mild aortic regurgitation, and mitral regurgitation.

**Figure 2:**
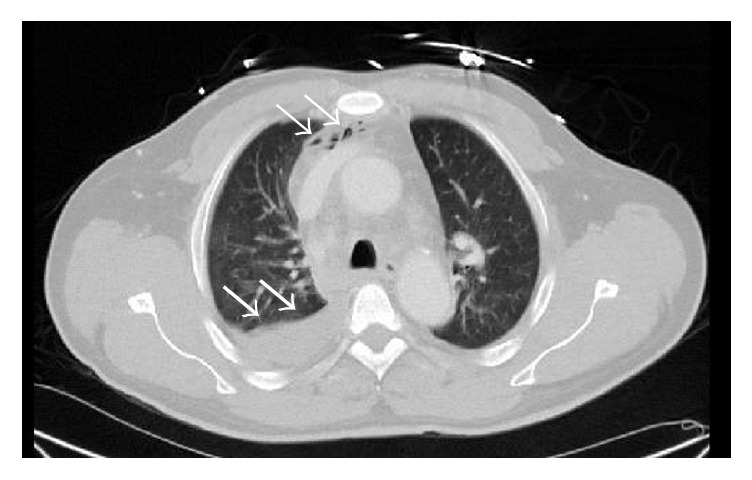
CT chest without contrast showed pneumomediastinum at the right superior mediastinum as shown by the white narrow arrow. The white thick arrow shows moderate right pleural effusion with 80 Hounsfield units suggestive of internal hemorrhage/proteinaceous content.

CT scan of chest revealed pneumomediastinum with mediastinal hematoma as seen in Figure 2.

## 4. Treatment, Outcome, and Follow-up

The patient presented to emergency room with acute nonradiating midsternal chest pain with evolving changes in anterior leads in EKG and a CODE STEMI was called for emergent cardiac catheterization as myocardial infarction is the most common and life-threatening condition that was needed to be ruled out in his EKG with ST elevation. Patient received aspirin and ticagrelor prior to catheterization. Coronary angiography showed normal coronary arteries but with mild global hypokinesis of the left ventricle that was already known even 6 months prior to this admission. Patient continued to have chest pain which was unrelieved by Nitroglycerin, while serial troponins remained negative and hence computed tomography of chest was done which revealed pneumomediastinum with mediastinal hematoma, likely a consequence of his unsuccessful attempted thrombectomy of his right brachiocephalic pain. Patient received topical as well oral pain medications for symptomatic relief. He did not require any surgical intervention as there was no collapsed lung and the volume of the pneumomediastinum can be absorbed by the body. He remained hemodynamically stable and was eventually discharged home with further follow-up with vascular surgery.

## 5. Discussion

Chest pain accounts for around 6 million annual visits to emergency department (ED) in the United States (US). Patients with EKG changes, characteristic symptoms, risk factors, and strong family history undergo emergent cardiac catheterization. However, in around 26% patients who underwent coronary angiograms, coronary arteries were found to be normal. In those patients, the presence of other noncardiac etiologies of acute chest pain needs to be assessed [1–4].

Pneumomediastinum is a rare condition described as abnormal presence of air or any other gas in the mediastinum. It can be spontaneous (primary) or secondary. Spontaneous pneumomediastinum is usually due to either infection or underlying lung conditions such as COPD or asthma [5]. Activities that cause increase in intrathoracic pressure such as Valsalva, coughing, and vomiting have also been reported to cause cases of spontaneous pneumomediastinum [6]. Secondary pneumomediastinum is usually a result of anatomic manipulation of mediastinal structures ranging from dental/endoscopic procedures [7], cardiac catheterizations, and endotracheal intubations to surgical interventions involving the thorax and abdomen. Chest or neck trauma causing tracheobronchial tree disruption has been reported to cause severe secondary tension pneumomediastinum. Pneumomediastinum secondary to injury of major venous vasculature at the time of central line placement has also been reported [8]. We present our case of secondary pneumomediastinum which resulted due to the manipulation of the right brachiocephalic vein, mimicking the signs and symptoms of a STEMI.

Apart from chest pain, patients with pneumomediastinum may also present with cough, shortness of breath, or changes in voice. Examination may be needed with Hamman's crunch or crepitations along the suprasternal notch. In terms of ECG findings, pneumomediastinum presents in various ways such as electrical alternans, T-wave inversions, poor R-wave progression, and low voltage QRS other than ST elevation [9, 10].

This is a rare case of secondary pneumomediastinum mimicking STEMI. The presence of other causes of chest pain in patients with normal coronary angiograms should be investigated. Care should be taken to ensure detailed history taking in regard to recent procedures involving the neck and thorax.

## Additional Points

*Learning Points/Take-Home Messages.* In patients who have persistent chest pain with normal coronary arteries with cardiac catheterization, history of any thoracic invasive procedures should give rise to suspicion for pneumomediastinum as a differential diagnosis in patients who present with EKG finding of ST elevation.
